# Intensity Analysis Method of Acoustic Emission Signals for the Damage Monitoring of Post-Tensioned Concrete Beams

**DOI:** 10.3390/ma17163981

**Published:** 2024-08-10

**Authors:** Ezio Bruno, Giuseppe Scionti, Luigi Calabrese, Edoardo Proverbio

**Affiliations:** Department of Engineering, University of Messina, Contrada di Dio, Sant’Agata di Messina, 98166 Messina, Italy; ezio.bruno@unime.it (E.B.); pepi_scionti@hotmail.com (G.S.); eproverbio@unime.it (E.P.)

**Keywords:** acoustic emission, corrosion, damage, post-tensioned concrete, intensity analysis

## Abstract

Acoustic emission (AE) is well suited for the real-time monitoring and detection of damage in reinforced concrete structures. In this study, loading/unloading cycles up to failure were applied on three different full-scale beams, each with varying defect morphologies. An intensity analysis method was employed to assess the damage sensitivities of the defective structures under stress conditions. Specifically, the calm ratio, load ratio, severity, and historical index were identified as statistical parameters that can provide global information on the damage level. Consequently, they can be easily used as damage evolution indexes for reinforced concrete structures. Correlations between these parameters were investigated to better discriminate between their potentials and identify critical levels that might not be evident using parametric analysis. The AEI chart helps locate damaging areas, aiding focused repairs. For defected beams with broken strands, at low load, HI and SI fall in zone B (damage detected). At cycles 4 and 6, with significant deflection, they fall into a critical zone, E (severe damage). Comparing post-tensioned beams revealed defects correlating with damage susceptibility. B3 beams with diffused defects displayed high activity at higher loads. Applying a load–calm ratio chart, initial minor damage worsened progressively. Severe damage was prominent in defective B2 and B3 beams, reaching zone 3. The variation in the acquired parameters over time can then be considered as an affordable and reliable indicator of damage progression.

## 1. Introduction

In civil engineering applications, such as building and bridge construction, post-tensioning or pre-stressing is a popular technique that allows for the design of remarkably slender yet highly functional structures [1]. This widespread adoption stems from the ability to create efficient designs that utilize less material while maintaining exceptional strength and performance [2]. While post-tensioning offers clear structural advantages, its long-term performance can be difficult to predict. This is because the actual lifespan of a post-tensioned structure is heavily influenced by environmental actions throughout its service life [3]. These actions can include climate variables (e.g., temperature, humidity, rain) or an aggressive environment (e.g., exposure to marine environment, deicing salts, and polluted atmospheres). Recognizing this challenge, researchers investigated real-world examples of premature failures and degradation in pre- and post-tensioned structures, specifically focusing on situations where localized attacks compromised the integrity of the tendons or surrounding concrete [4,5,6,7].

There is a growing need for a dependable and affordable way to track and evaluate how damage develops in reinforced concrete structures, especially pre- and post-stressed concrete [8]. This need is driven by two key factors: First, existing structures are becoming older, which can lead to a worsening of their condition through various deterioration processes. Second, the load-bearing demands placed on these structures are constantly rising, making it even more crucial to ensure their reliability and performance. The development of structural health monitoring (SHM) techniques is therefore of great concern in the field of civil engineering [9,10,11,12,13].

Acoustic emission (AE) has recently attained growing awareness as a monitoring approach with which to assess the stability and safety of reinforced concrete structures [14,15]. This technique allows for the assessment and monitoring of these structures by detecting and analyzing sound waves generated by movement or damage within the materials.

While the acoustic emission technique holds promise for monitoring the health of civil engineering structures, its implementation faces several challenges. The complexity of structures, the inherent variability in concrete materials, and the attenuation of an AE signal as it travels through a structure all present hurdles [16]. Additionally, environmental noise can further mask the subtle acoustic emissions [17,18]. However, recent years have seen significant advancements in the analysis and interpretation of AE data, making it a more viable tool for civil engineers [19,20,21,22].

Multivariate statistical approaches have been used to suitably cluster the complex AE database and identify features that are important for understanding the main damage mechanisms in concrete [23,24]. Techniques like principal component analysis (PCA) and artificial neural networks (ANNs) have been applied to this problem and show promising results for accurately classifying the type of damage based on the AE signal [25,26].

While these numerical methods offer a powerful approach, they have two key drawbacks. Firstly, they are not user-friendly, meaning they require specialized knowledge to be set up and interpret the results. Secondly, defining and differentiating the clusters within the data demand a strong scientific background in the specific topic.

Considering these limitations, researchers are exploring alternative methods for assessing structural damage under load using AE data. These methods prioritize user friendliness and aim to be more accessible. In particular, statistical parameters like the calm ratio, load ratio, severity index, and historical index offer valuable insights into the level of damage a structure has sustained. Due to their relative simplicity, these parameters can be readily employed as damage-triggering and growth indicators in various concrete building components [27,28]. 

This research explores the potential of acoustic emission for the evaluation of the cumulated damage of PT reinforced concrete beams. A dual-test setup was adopted, aiming to achieve AE source localization and AE waveform acquisition. AE waveforms are fundamental for identifying and discriminating source type and relating it to the dominant damage mechanism.

To understand the damage evolution in a post-tensioned concrete beam, continuous AE recording was conducted during loading and unloading cycles until the beam reached failure. The study investigated three beams: an undamaged one, a beam with a broken strand due to corrosion (B2), and a beam with induced microbiological corrosion on steel strands (B3). The processed AE signals are the primary data source for this study. After filtering by source location, the signals were analyzed using the intensity analysis (IA) method to assess damage accumulation within the beam. 

## 2. Materials and Methods

Acoustic emission monitoring was performed on post-tensioned concrete beams through cyclic loading and unloading conditions. The beams were characterized by a length of 6.30 m and a rectangular cross-section with dimension 0.40 m width and 0.25 m thickness. Each beam was constructed from a concrete mixture designed for a compressive strength of 55 MPa. The mix included 430 kg/m^3^ of ordinary Portland cement (OPC) and was strengthened with four 18 mm steel bars and post-tensioned with four 7-wire strands measuring 6/10 inches in diameter. Three different beams were investigated:

- Beam B1: Undamaged beam.
- Beam B2: Broken strand. The grouting process fully encapsulated the tendon with the exception of a localized corrosion pocket measuring 25 cm in length, situated in the central region of the beam. In this central area, a corrosive solution was applied until failure of the strand occurred.
- Beam B3: Microbiological-induced corrosion. The tendon was ungrouted. A grease contaminated by fungi was injected into the duct in order to promote corrosion on the steel strand as a consequence of grease degradation.

All of the beams were loaded in a three-point bending test set-up. Load has been progressively applied by a hydraulic actuator (maximum capacity of 1000 kN of force and 700 mm of displacement) pushing on a load distribution block made up of two steel plates separated by two cylinders. Details concerning the applied beam loading cycles are reported in Table 1.

**Figure 1:**
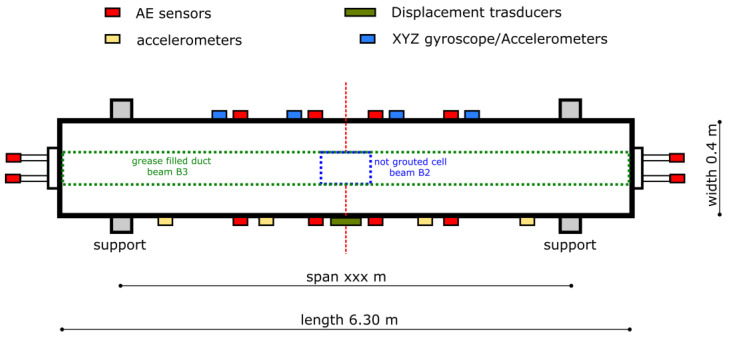
Scheme of sensorized post-tensioned concrete beam.

AE signals have been recorded by an AMSY-6 Vallen instrument (Vallen Systeme GmbH, Wolfratshausen, Germany), equipped with 12 high-sensitive AE sensors, to obtain both source localization and AE waveforms. Continuous recording was carried out to acquire all of the AE hits that occurred during the whole loading/unloading cycles. Sensors were located homogeneously along the beam length (see red squares in Figure 1). Accelerometers and gyroscopic as well as displacement sensors (not discussed here) were also applied on the structure to acquire a more effective signature of beam deformation during the test. A scheme of a sensorized post-tensioned concrete beam is depicted in Figure 1. In the figure, displacement, accelerometer, and AE sensors’ locations are denoted. Furthermore, the locations of the not-grouted cell for beam B2 and the grease-filled duct for beam B3 were depicted. 

## 3. Intensity Analysis Method

**Figure 2:**
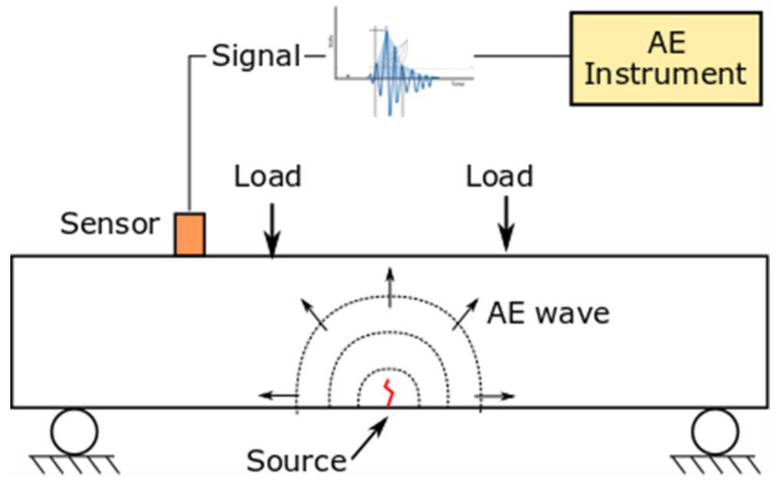
Scheme of the AE principle.

Acoustic emission will produce transient waves as a result of both the generation and the propagation of cracks within the material at increasing loads. The propagation of these cracks creates rapid releases of energy that manifest as transient waves, detectable by AE sensors. This phenomenon is clearly illustrated in Figure 2, which depicts the underlying principles of the AE technique applied to a concrete beam subjected to stress conditions. The figure shows the evolution of an AE wavefront (dotted circles) starting from the AE source (the crack caused by applied flexural load schematized as a red, irregular line) towards the AE sensor (orange rectangle), which is able to detect the signal over time. Therefore, the figure visually represents how the AE method captures stress-induced acoustic signals, highlighting the relationship between the internal stress, crack dynamics, and the corresponding acoustic emissions in the material.

The intensity analysis (IA) method is a quantitative technique based on mathematical calculations to assess the structural integrity and deterioration level of a concrete structure by means of some damage indexes. It achieves this by determining two key values: the historic index (*HI*) and severity index (*SI*) [29]. In agreement with [30], this approach has been identified as a successful statistical method applicable for evaluating various materials, such as FRP, metal, and concrete, across different evaluation systems. The equations for *HI* and *SI* are shown in Equations (1) and (2), respectively [29,31]:

(1) $$HI=\frac{N}{N-K}\left(\frac{\sum_{i=K+1}^{N}S_{oi}}{\sum_{i=1}^{N}S_{oi}}\right)$$

(2) $$SI=\frac{1}{J}\left(\sum_{m=1}^{J}S_{om}\right)$$

*N* = number of AE hits up to time t. 

*Soi* = signal strength of the *i*^th^ hit. 

*Som* = signal strength of the *m*^th^ hit, where the order of m is based on the magnitude of the signal strength.

For concrete, *K* and *J* are parameters, according to [29,31], strictly related to *N*, the number of hits. 

Analogously for concrete material *J* = 0 for *N* < 50; *J* = 50 for *J* > 50. 

Therefore, the maximum *HI* and *SI* values can be plotted on intensity charts, where these parameters are on the X and Y axes, respectively. Depending on the combined magnitude of *HI* and *SI*, different damage levels of the structure can be argued. 

Furthermore, to obtain critical damage levels of post-tensioned concrete beams, specific AE-based parameters, the defined load ratio (LR) and calm ratio (CR), were determined. 

The method relies on the normalization of analyzing AE data and stress–strain gathered during repeated loading and unloading cycles [27]. A key parameter is the load ratio (LR), which compares the load when acoustic emission (AE) first appears during a cycle to the maximum load from the prior cycle. This ratio helps distinguish between two material behaviors: the Kaiser effect and the Felicity effect. If the LR is close to 1, this suggests that the Kaiser effect occurs. This indicates that concrete structure behavior is influenced by the previously reached maximum load level. In this case, the onset of AE activity is quite similar to the maximum load of the previous loading cycle. In this case, the material can be considered structurally stable and no significant damage phenomena are evolving. For an LR < 1, the material shows the so-called Felicity effect [32]. In this case, the load at the onset is acquired before the previously achieved maximum load cycle. This condition is related to a high sensitivity of the structure to damage activation and propagation. An LR of 0.9 was identified as the threshold between the Kaiser and Felicity effects [27]. Instead, the CR parameter is defined as the ratio between the AE events recorded during unloading to the AE events for the whole loading–unloading cycle. 

## 4. Signal Denoising

### 4.1. Source Localization Method

This section introduces a general mathematical model that estimates the three-dimensional (3D) location of an emitter using information from the AE sensors, and it was defined according to the localization approach reported in [33]. 

In the study, the signal used to detect AE source is first defined. The signal is emitted by the source, travels through the medium, and reaches receivers (AE sensors) at different times due to varying distances. The time of travel (*ToT*) is the signal’s travel time. The time of emission (*ToE*) marks signal emission. The time of arrival (*ToA*) is the receiver detection time. The *ToT* can be calculated from the *ToE* and *ToA*:

*ToT* = *ToA* − *ToE* (3)

The distance between the emitter and receiver, denoted by *d_ER_*, can be expressed based on the *ToT* and the speed of the wave in the medium (*c*):

*d_ER_* = *ToT* · *c* (4)

To pinpoint the AE source, at first it is necessary to know the position, (*x_i_*, *y_i_*, *z_i_*), of each receiver in a Cartesian coordinate system. The source location is denoted by (*x_e_*, *y_e_*, *z_e_*). *d_ER__i_* is defined as the distance between the emitter and the i-th receiver:

(5) $$d_{{ER}_{i}}=\sqrt{{\left(x_{i}-x_{e}\right)}^{2}+{\left(y_{i}-y_{e}\right)}^{2}+{\left(z_{i}-z_{e}\right)}^{2}}$$

Equation (5) establishes a solvable system of nonlinear equations with three unknowns, represented by *x_e_*, *y_e_*, and *z_e_*. To locate the source, a system of equations with *m* equations and *n* unknowns was solved based on the Newton–Raphson method using a Python script. The program’s settings include a maximum of 4000 attempts, an error tolerance of <0.001 m, and no limit on function evaluations.

### 4.2. Denoising of the AE Dataset 

The denoising process of the acoustic emission signals involved applying the localization approach. This effectively filtered out any events not originating within the defined span length of the concrete beams. These extraneous signals, identified as noise, were excluded from the subsequent data analysis to ensure the accuracy and reliability of the results. By implementing this filtering criterion, it can be possible to focus on the main relevant acoustic emission events occurring within the designated area of interest. This approach is able not only to enhance the quality of the data analysis but also contributes to a more comprehensive understanding of the structural behavior under investigation. 

**Figure 3:**
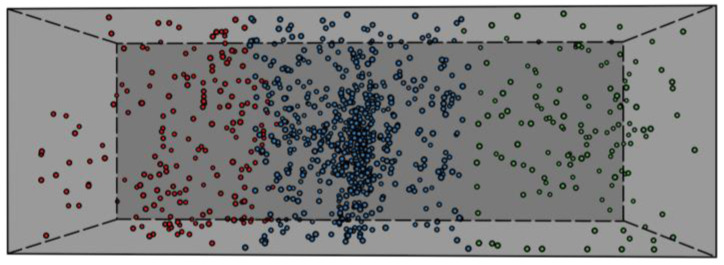
Distribution of the source locations of AE events acquired for the B2 beam during cycle 1.

For reference, Figure 3 shows the distribution of the 3D sources of the acoustic events detected for beam B2 following the application of the localization model at load cycle 1. 

In Figure 3, the AE events were grouped according to their sources, with distinct marker colors reflecting their placement to the left, middle, or right of the applied load. Notably, a prominent cluster resides beneath the applied load, constituting the most densely populated region within the dataset, indicating the most critical region. 

All events originating within the beam’s load region were considered valid, while those outside were excluded as noise from the AE dataset population, as they were not representative of real acoustic events. This approach was systematically implemented for each individual beam across all cycles in the study. By following this procedure, it was possible to effectively identify and remove around 25% of extraneous events that were not connected to any specific acoustic activity. This critical step helped to enhance the overall accuracy and reliability of the data collected, ensuring that only relevant acoustic events were analyzed. Furthermore, the application of this method allowed for a more suitable examination of the acoustic signals detected, leading to a comprehensive understanding of the underlying patterns and trends in the AE data.

## 5. Results and Discussion

### 5.1. AE Activity

**Figure 4:**
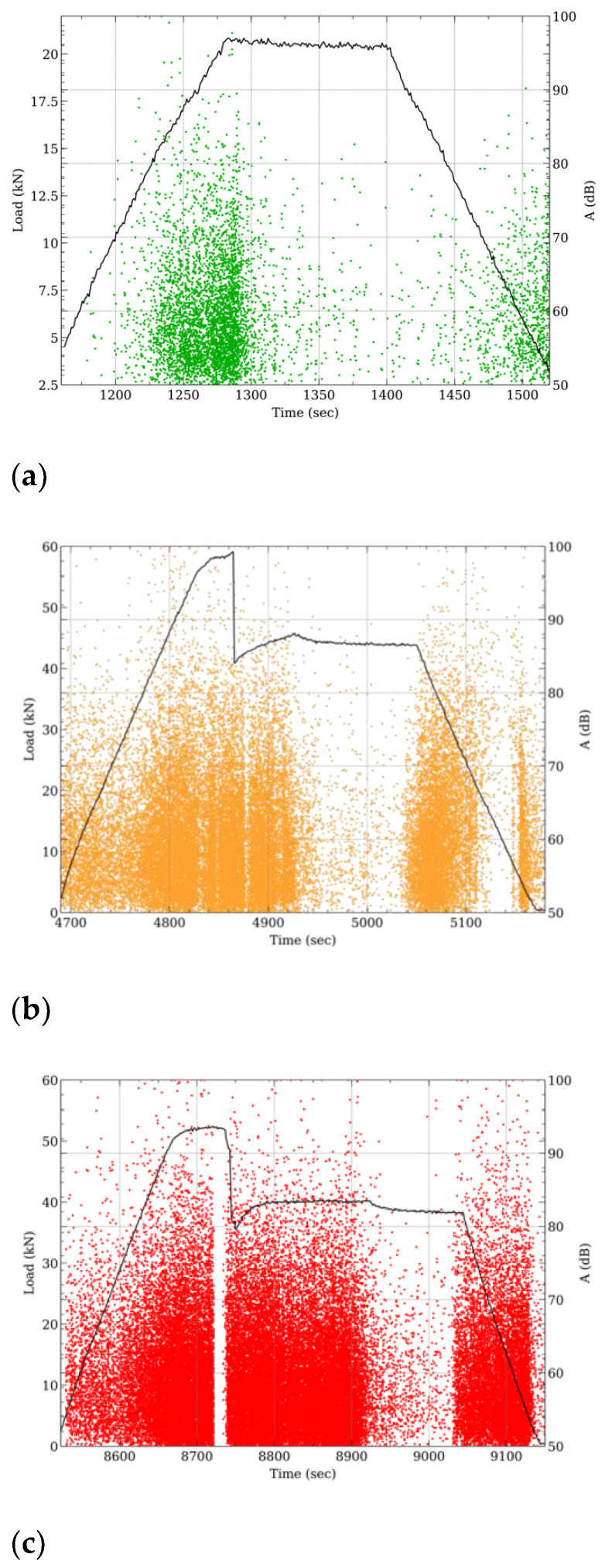
Amplitude of AE events and load evolution for three reference cycle set for the B2 beam: (**a**) 10 mm (cycle 1), (**b**) 60 mm (cycle 4), and (**c**) 100 mm (cycle 6) deflection.

Preliminarily, to assess the behavior of post-tensioned concrete beams under various loads, detailed plots illustrating the progression of acoustic events during load cycles could prove to valuable insights into damage evolution. These graphs, reported in Figure 4, showcase the amplitude distribution of AE events and load changes across different reference load cycles. Specifically, in Figure 4, the patterns for three key stages in the B2 beam’s deflection can be observed: (a) at 10 mm deflection (cycle 1), (b) during 60 mm deflection (cycle 4), and (c) at 100 mm deflection (cycle 6). These visuals offer valuable insights into how the beam responds to the applied loads at distinct steps during the cycle.

In detail, at varying loading cycles, the following considerations can be argued:

- Cycle 1: At low maximum deflections (during cycle 1—Figure 4a), the stresses affecting the beam are quite limited, resulting in minimal acoustic activity being generated. This indicates a fairly elastic behavior of the beam under these specific conditions. An observation of the amplitude pattern throughout the varying loads showcases the scarce acoustic events occurring in the initial stages. Notably, as the load increases within the force range of 14–20 kN, there is a marked increase in acoustic activity, with events reaching amplitude levels above 80 dB. Interestingly, as the load stabilizes, the occurrence of acoustic events diminishes significantly in both intensity and event frequency, suggesting that the beam behaves inertly and does not exhibit any critical structural vulnerabilities in such stress states. Moreover, during the unloading phase, there is a gradual relaxation of the stresses, evidenced by a brief episode of low-intensity acoustic activity, further validating the beam’s stable response throughout the loading and unloading processes.
- Cycle 4. Concerning cycle 4 (Figure 4b), where an intermediate maximum deflection of 60 mm is applied, a noticeable distinction can be observed regarding the progression of acoustic events throughout the entire loading cycle. Similar to the pattern observed in cycle 1, the peak of acoustic activity coincides with both the loading and unloading phases; however, unlike cycle 1, cycle 4 exhibits some distinct characteristics that differentiate its AE and mechanical behavior. Right from the initial stages of the loading cycle (characterized by minimal deflection levels), the structure exhibits a substantial increase in acoustic activity. This increased acoustic activity is evident not only in the higher number of acquired acoustic events but also in their magnitudes. While the stabilization phase aims to achieve a mechanically steady state, there is a possibility of encountering further deviations from the desired behavior. This is especially concerning in this particular region, as the structure itself is not structurally stable. In fact, a clear collapse and abrupt reduction in load-bearing capacity is observed after about 20 s from the beginning of the maintenance phase. This critical failure occurs at an applied load level of 58.5 kN, highlighting the limitations of the structure. This phase coincides with the structural failure of the beam, resulting in a significant loss of its load-bearing capacity. This period of mechanical instability is accompanied by intense and continuous acoustic activity, with recorded AE events reaching high amplitudes. Notably, this activity persists even after the load collapses, suggesting ongoing damage settlement, potentially involving further coupled crack propagation followed by a potential arrest. Following the stabilization of the beam, there is a cessation of any additional acoustic events as the maintenance phase continues. This lack of further AE events remains consistent throughout the remaining duration of this phase. During the unloading phase, as acoustic activity returns, there is a notable re-emergence of AE events in the impacted area. This phase is marked by additional movement within the damaged region as the existing cracks begin to close. Such relocation within the material leads to the genesis of new acoustic events, some of which possess a significant level of intensity. The progression of this phase indicates a reactivation of dynamic processes within the material, potentially signaling shifts in the structural integrity and acoustic responses of the system under study.
- Cycle 6. The beam, which in cycle 4 had weakened due to a broken strand, demonstrated unexpected residual strength during the subsequent cycles, even as it experienced larger deflections. Notably, in cycle 6 (Figure 4c), where the deflection increased by 40% compared to cycle 4, the beam managed to sustain a similar load (approximately 55 kN and 58.5 kN for cycle 6 and cycle 4, respectively), hinting at a considerable loss in bending stiffness. Consequently, the beam’s structural integrity was quite compromised, leading to instances of mechanical instability characterized by load fluctuations and drops during the stabilization phase. These fluctuations were likely attributed to further strand breaks or slippage at the fracture points within the beam [34]. The resulting damage was not isolated but spread throughout the beam, triggering multiple acoustic events across a significant portion of the cycle. This widespread acoustic activity served as a clear indication of the ongoing and extensive structural damage within the beam.

**Figure 5:**
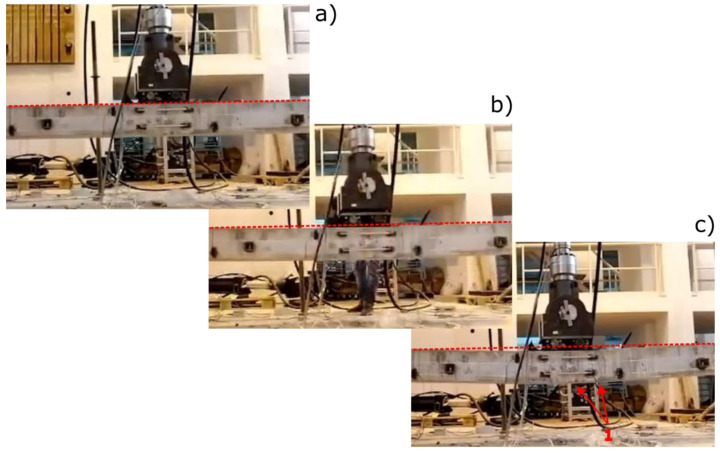
Images of damage level in the B2 beam at the end of (**a**) cycle 1, (**b**) cycle 4, and (**c**) cycle 6.

Further interesting information can be argued by evaluating, in Figure 5, the images that depict the damage level in the B2 beam at the conclusion of cycle 1, cycle 4, and cycle 6 (referred to as Figure 5a, b, and c, respectively). These images provide valuable insights into how the beam’s structural integrity evolves over time and the impact of repeated cycles on its overall condition. Via the analysis of the images from each cycle, trends, variations, and potential areas of concern that may arise as the beam undergoes cyclic loading can be detected. Exploring the changes in damage patterns and severity between cycles can offer valuable data regarding the beam’s performance and enable a better understanding of its durability and long-term reliability.

Although post-tensioned concrete beams have high flexural resistance, when subjected to increasing flexural loads they showed significant and progressive damage phenomena. Initially, at low load levels, the structure remains elastic (cycle 1, Figure 5a). No significant cracks have been detected on the beam, indicating the good structural stability of the structure at these stress levels. Very small micro-cracks, visible only by deep visual investigation, might appear in the concrete’s tension zone as a sign of minor stress. No evidence of permanent deflection can be identified when the applied load is released (horizontal dotted red line, Figure 5a).

As the deflection increases up to 60 mm (cycle 4, Figure 5b), the micrometric cracks widen and become visible to the naked eye (macro-cracks). As the load increased, these cracks propagated perpendicular to the axis of the beam, toward the applied load direction, increasing the deformability of the flexed section. These cracks affect the stiffness of the beam and lead to a reduction in its load-bearing capacity [35].

The concrete’s ability to resist stress weakens, forcing the pre-stressing tendons to carry a greater load. The steel reinforcement comes into action, supporting a larger amount of the applied load. As the load progresses, cracks also begin to appear in the compression zone, near the application of the flexural load. The beam is also characterized by a slight permanent deflection when the load is released (horizontal dotted red line, cycle 4, Figure 5b). In this case, the ability of the concrete to withstand compressive forces is compromised. A further increase in load leads to the rupture of the tendon and the consequent collapse of the load, as was identified in Figure 5b, relating to cycle 4.

During cycle 6, involving more intense deformation states (100 mm deflection, Figure 5c), the concrete cover separates from the post-tensioned reinforcement bar (point 1 in Figure 5c). This phenomenon is an additional indicator of significant permanent damage of the structure. Simultaneously, the increased deformation increases the relative slip between the cement matrix and steel reinforcement, inducing delamination at the interface and slippage. These factors amplify the widening of the damage zone, diminish the stiffening as well as concrete resistance action provided by the steel strand, and ultimately lead to the further rupture of other strands and a significant collapse of the beam’s mechanical strength. As confirmation of the large damage suffered by the post-tensioned concrete beam, a large permanent deflection when the load is released can be identified after cycle 6 (horizontal dotted red line, Figure 5c).

### 5.2. Intensity Analysis

**Figure 6:**
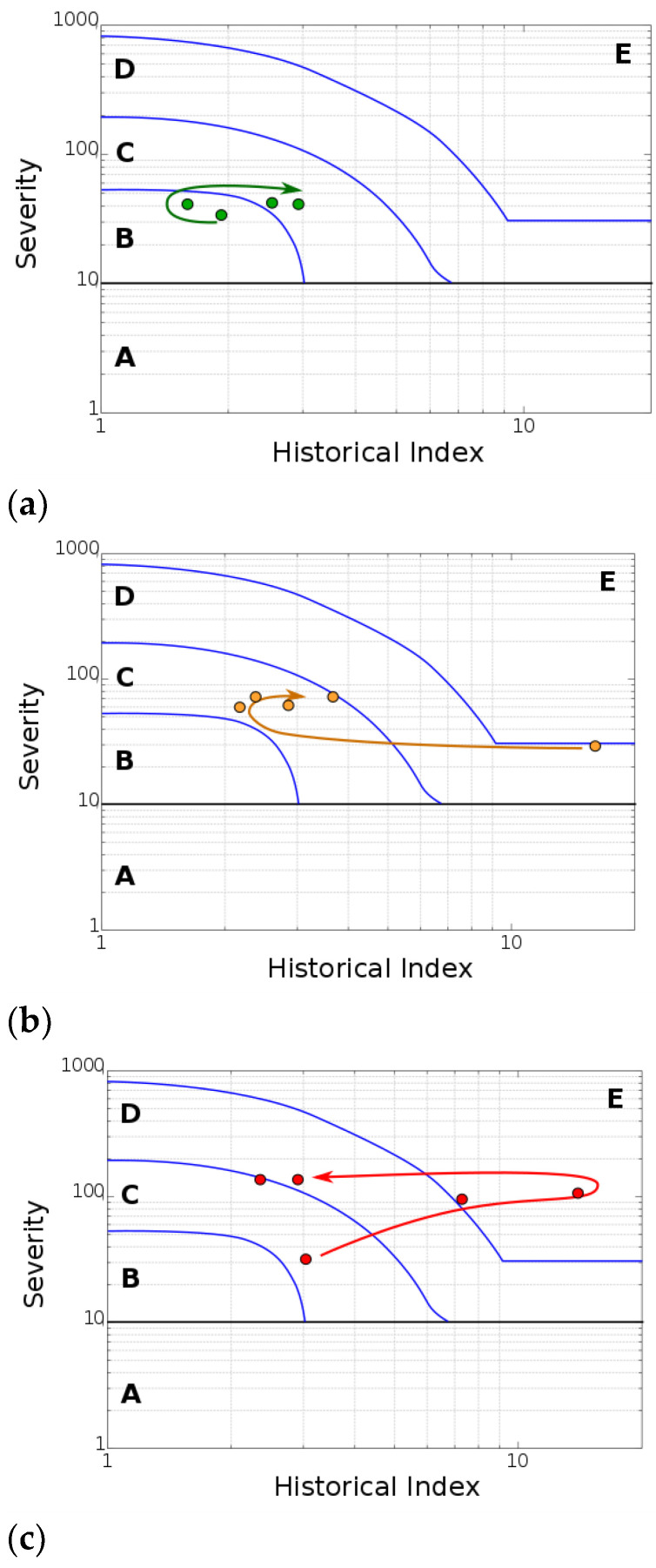
Absolute energy intensity chart for the three-reference-cycle set for the B2 beam: (**a**) 10 mm (cycle 1), (**b**) 60 mm (cycle 4), and (**c**) 100 mm (cycle 6) deflection.

All waveform parameters were analyzed by using the IA method. For each beam, the data signals were evaluated for each specific loading cycle. This analysis involved a cycle-by-cycle evaluation of the data signals for each individual beam. Figure 6 shows the absolute energy intensity (AEI) chart specifically for three reference cycles pertaining to the B2 beam. 

This chart incorporates a plot of both the severity index and the historical index. By analyzing these plotted values, different intensity zones can be identified within the chart. These zones, in turn, provide valuable insights into the structural significance of the emissions detected [36]. A comprehensive description of these zones (A through E) is provided in Table 2.

At low loading levels (Figure 6a, referring to cycle 1), the HI and SI values measured at progressively longer cycle times fall within zone B or near zone C on the map. This indicates that minor surface defects can be detected; however, no significant structural damage is evident at this stage [37,38]. 

As the load levels increase (Figure 6b, referring to cycle 4), all zones within the beam progress to zones C and D [39]. The higher stress levels cause major damage to the post-tensioned concrete beam. The AE data signals appearing in zone D of the intensity chart mean significant damage to the beam structure, potentially progressing towards a state of structural instability (zone E) [40]. This loading stage also witnessed a sudden decrease in load, likely due to the propagation of large cracks and a permanent deflection status. Mandatory inspection and evaluation of these defects are necessary.

Finally, at high load levels, the loading cycle segments reach extremely high intensity levels, as shown in the intensity chart of Figure 6c. At this point, the zones are considered to be in a critical failure zone, indicating unsafe conditions. From a structural standpoint, the beam requires immediate shutdown and a thorough follow-up inspection with ongoing monitoring.

The intensity plots in Figure 6 reveal a direct correlation between the total energy released (absolute energy) and the severity of the damage (damage grading level) progressively inflicted on the material during the loading test. This suggests that absolute energy can be a reliable method for visually assessing the extent of damage on the macroscopic level. Furthermore, by analyzing the distribution of this absolute energy throughout the structure (represented by the AEI chart), the areas experiencing the most significant damage could be pinpointed, allowing for the targeted identification of structural weaknesses in concrete structures.

Further interesting considerations can be evaluated by comparing the AEI charts for three post-tensioned concrete beams at varying starting defect levels. All points were carried out based on the number of loading cycles. The total amount of cycles changes depended on the tested beam, considering that some elements are more sensitive to premature fractures at lower loading conditions compared to other ones. 

Interestingly, beams characterized by initial artificial defects exhibit a heightened susceptibility to damage initiation and subsequent propagation. A prime example is beam B2, which contains a localized strand failure. The chart reveals a significant number of AE events at high intensity levels. This signifies the presence of substantial defects that emerged and evolved at relatively low stress levels. This vulnerability is likely caused by the localized corrosive attack, which predisposed the structure to further damage. One particularly noteworthy point on the chart for B2 is the event at cycle 4, which falls within the highest severity level (E).

Similar considerations can be argued for the B3 beam, characterized by a diffused defect (ungrouted duct) and light-induced microbiological corrosion on steel strands. The absence of a grouted duct clearly modifies the beam response at a high load, promoting the formation of highly acoustically active defects, as evidenced by points C4, C5, and C6, located in zone E.

### 5.3. Load–Calm Ratio

**Figure 7:**
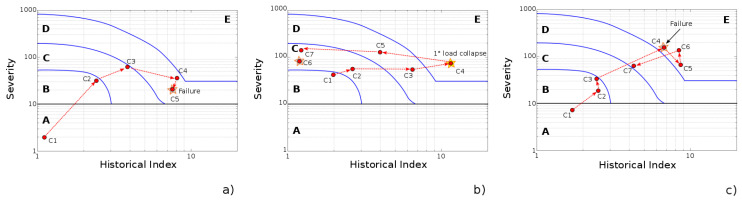
Specimen damage evolution by absolute energy intensity chart at increasing cycles for the (**a**) B1, (**b**) B2, and (**c**) B3 beams.

**Figure 8:**
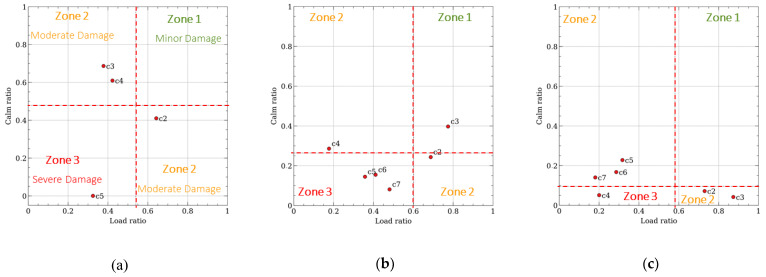
Beam damage evolution by load and calm ratio charts in the (**a**) B1, (**b**) B2, and (**c**) B3 beams.

Building upon the data presented in Figure 7, Figure 8 delves deeper into the relationship between the load ratio (LR) and the calm ratio (CR) for all of the tested beams throughout their entire loading cycles [41]. These charts reveal three distinct zones, each corresponding to a specific level of damage according to the established LR-CR classification system. In the comprehensive system implemented, the classification is as follows: zone 1, which indicates minor damage that might require minimal intervention; zone 2, signifying a moderate level of damage that may necessitate more involved repair efforts; and, finally, zone 3, representing severe damage that would likely call for significant restoration work. Each specific zone is clearly outlined using intersecting dashed lines, which aid inspectors and engineers assessing structural integrity in terms of visual demarcation and easy identification. The observed variations in CR values between the beams is attributable to the discrepancies in the number of acoustic emission hits registered during the tests. Notably, the calm ratio is calculated based on the number of AE events recorded during both the loading/unloading phases, making it sensitive to the structural health of the material.

In the early stages of testing, the low magnitude values suggest that there was minor damage to the material, even though no visible cracks were present. This observation is consistent with the findings from the classification process. As the testing progressed, a pattern emerged across all of the beams, where there was a peak in the calm ratio and a corresponding minimum in the load ratio during the intermediate cycles, signifying a state of significant damage. Toward the latter stages of testing, a common trend surfaced, where the calm ratio continually decreased while the load ratio remained relatively stable. 

Further investigation into the specific damage phenomena observed in beam B3 revealed a progressive increase in the calm ratio, which is believed to be linked to the artificial defect—applied bio-corrosion greased strands. 

It is relevant to delve deeper into multivariate acoustic emission analysis and effectively integrate this information with complementary sensing techniques. By doing so, researchers can enhance the study of damage in structures similar to this one, ultimately adding significant value to the investigation. This enhanced understanding can greatly contribute to the comprehensive assessment of structural integrity and aid in devising effective strategies for mitigating the potential risks associated with damage to this specific type of structure.

## 6. Conclusions

The present paper addresses an examination of the applicability of the AE technique for the SHM of post-tensioned concrete beams. Real-time AE acquisition has been carried out to attain the AE hits originated during both the loading and unloading cycles up to the beam fracture on an almost-real-scale post-tensioned concrete beam (length of 6.30 m and rectangular cross-section with a width of 0.40 m and thickness of 0.25 m). In particular, the intensity analysis method was applied to assess the damage severity on post-tensioned beams. Finally, the AE signal strength data indicate this approach effective in comparatively evaluating damage evolution stages on post-tensioned concrete beams differing in the presence of artificial defects. 

The distribution of this energy on the AEI chart helps pinpoint specific locations experiencing significant damage, enabling targeted repairs and the improved monitoring of concrete structures. For a broken strand beam (B2), at a low applied load (cycle 1) the HI and SI values fall within zone B (damage detected). At cycle 4 and cycle 6, characterized by a large deflection (60 mm and 100 mm, respectively), the HI and SI values fall within a more critical, E, zone (severe damage) in the AEI plot. Comparing the AEI charts of the post-tensioned concrete beams with varying initial defects revealed a clear correlation between defect level and damage susceptibility. Beams with an artificial defect, like B2, with localized strand failure, showed a significant increase in AE events at lower stress levels, indicating earlier damage initiation and propagation. This highlights the vulnerability introduced by such defects. Similar trends were observed in B3 with diffused defects, where the absence of grouting and microbiological corrosion resulted in highly active defects at higher loads. Similar considerations were argued when applying a load–calm ratio chart. Initial cycles showed minor, invisible damage; further analysis revealed a progressive worsening throughout the later cycles. Zone 3, referring to severe damage, is largely reached for defected B2 and B3 beams. 

In summary, the results evidenced that the absolute energy approach can be considered a suitable identifying method with which to discriminate the damage grading level that gradually evolves during the applied loading test conditions topologically.

## Figures and Tables

**Table 1:** Beam loading cycles.

Load Stabilization (sec.)	Deflection (mm)	Cycle
300	10	1
50	20	2
50	40	3
60	60	4
120	80	5
100	100	6
400	150	7

**Table 2:** Significance of intensity zones. Adapted from [29].

Intensity Level	Structural Significance
A	No damage. Not relevant acoustic emission.
B	Damage detected. Typically, small-crack-triggering under applied stress.
C	Minor damage. Any detected defects necessitate additional assessment. This assessment could be based on data analysis or performing supplementary non-destructive tests.
D	Major damage. Relevant defects occur that require a closer look.
E	Severe damage. Large and extensive defects need instant shutdown and a thorough inspection.

## Data Availability

The original contributions presented in the study are included in the article, further inquiries can be directed to the corresponding author/s.
